# Cutaneous larva migrans in the city

**DOI:** 10.1002/ccr3.3262

**Published:** 2020-09-29

**Authors:** Aruna S. Khan, Ahmad Al‐Awadi, Floyd B. Willis, George G. A. Pujalte

**Affiliations:** ^1^ Department of Family Medicine Mayo Clinic Jacksonville Florida; ^2^ Florida State University Tallahassee Florida

**Keywords:** cutaneous larva migrans, parasitic disorders, tropical diseases

## Abstract

Cutaneous larva migrans is typically reported in tropical and subtropical regions of the world. However, cutaneous larva migrans cases are spreading and should now be recognized even in urban, nontropical settings.

## Graphical abstract

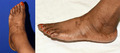

## 1 CASE REPORT

A 66‐year‐old woman presented with a pruritic rash on her left foot for the past 1 month. Blisters appeared on the dorsum of her foot within a few days of scratching, spreading longitudinally along the initial lesions. They were not associated with pain, swelling, or discharge. She denied any fever, chills, weight changes, other skin lesions, recent travel, or trips to the beach. She applied rubbing alcohol and witch hazel without relief. She did not own pets, but her neighbor's cats were frequently in her yard. She enjoyed gardening, wore clean socks and shoes, and seldom walked barefoot in her yard. She lived in Jacksonville, Florida.

**FIGURE 1:**
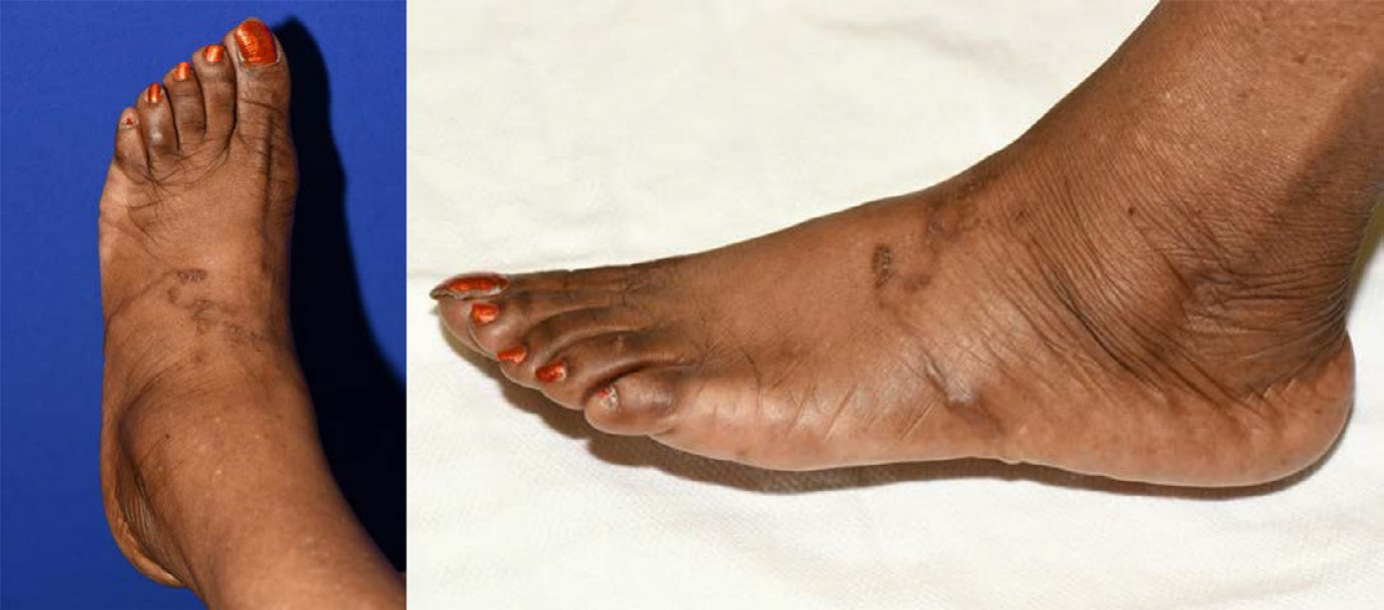
Serpiginous Skin Manifestation Noted on Patient's Left Foot. The patient reported that it originated from the dorsal midfoot and traversed to the inferior lateral border of the foot. Photograph taken on 13 October 2015 before ivermectin therapy

On examination, the rash appeared serpiginous (Figure 1), exhibiting burrows on the dorsum of her foot, extending to the plantar surface. Her foot was not erythematous, warm, swollen, or tender to palpation. No fluid‐filled blisters or vesicles were present. She was diagnosed with cutaneous larva migrans (CLM), referred to Dermatology, and treated with 200 µg/kg of ivermectin with resolution of symptoms within 1 week.

## 2 DISCUSSION

Hookworm‐related CLM is a human parasitic self‐limited dermatosis caused by the invasion and migration of animal hookworm larva into the epidermis. CLM is usually found in the tropics and subtropical areas of the world where the temperatures and moisture are optimal for larva survival. In the United States, patients may present with this condition after travelling to endemic areas: Caribbean, Southeast Asia, Central America, or South America.^1^ However, CLM can also thrive in the warm, moist, sandy regions of Florida and the Gulf Coast.^2^ While the adult form lives in the intestine of dogs and cats, the eggs are shed in fecal matter. Once the eggs hatch in soil, the larvae progress into the infective third‐stage until they locate a host.^2^ Our patient likely obtained her condition from gardening in soil contaminated with cat fecal matter.

Patients initially experience paresthesia as larvae enter the skin, followed by an asymptomatic dormant stage or the symptomatic larva migration phase. The entrance site can be anywhere on the skin, including fissures, sweat glands, or follicles.^3^ After the dormant phase, patients experience pruritus that may cause sleep disturbances.^1^ Without treatment, the sequelae of CLM include secondary bacterial infections, Lӧffler's syndrome,^4^ and eosinophilic enteritis. Febrile CLM may indicate concurrent human immunodeficiency virus infection.^5^

Patients may also present with atypical features.^6^ The eczematous type manifests as circular inflammatory plaques covered by a vesicular, scaly, crust‐like appearance; short tracks may be visible. Alternatively, tinea pedis‐like type include vesicles, scaling, and crusts without tracks. The bullous type presents as a large, oval, clear serous‐filled blister along a track. The follicular type is characterized by erythematous papules that may be topped by vesicles or pustules.^6^ Clinicians should maintain a broad differential including allergic/irritant contact dermatitis, tinea pedis, larva currens, scabies, and photoallergic dermatitis.

Treatments of CLM include antihelminthic agents including ivermectin, oral albendazole, mebendazole, or topically administered thialbendazole. A single oral dose of ivermectin (200 µg/kg) effectively kills the larva; refractory cases require a second dose.^1^ Ivermectin has superior outcomes compared with albendazole; however, repeated doses with albendazole are a good alternative in countries where ivermectin is unavailable.^1^ After treatment, the pruritus usually resolves within 24‐48 hours, and the lesion resolves after 1 week,^7^ as demonstrated with our patient. Prevention strategies include testing and treating infected cats and dogs, reporting stray animals, promptly removing animal fecal matter from areas of human activity,^8^ and wearing proper footwear.^1^

In summary, cases of CLM have been reported in the tropics and subtropical regions of the world. This case brings awareness to the emergence of CLM in the city as our patient most likely developed the skin lesion from being at home gardening, without any recent travel history.

## CONFLICT OF INTEREST

Dr Pujalte occasionally receives speaker fees from American Medical Seminars and occasionally has travel expenses paid by USA Taekwondo for sports coverage. The authors have no conflicts of interest to report related to this work.

## AUTHOR CONTRIBUTIONS

ASK and AAA: were involved in the manuscript writing, editing, and literature review of this case report. FBW: was involved in caring for the patient discussed, writing, editing, and reviewing the manuscript. GGAP: was involved in writing, editing, and reviewing the manuscript.
